# CKD-EPI creatinine-cystatin C glomerular filtration rate estimation equation seems more suitable for Chinese patients with chronic kidney disease than other equations

**DOI:** 10.1186/s12882-017-0637-z

**Published:** 2017-07-10

**Authors:** Xiao-Hua Chi, Gui-Ping Li, Quan-Shi Wang, Yong-Shuai Qi, Kai Huang, Qian Zhang, Yao-ming Xue

**Affiliations:** 1 Department of Nuclear Medicine, Nanfang Hospital, Southern Medical University, Guangzhou, Guangdong 510515 China; 2 Department of Endocrinology and Metabolism, Nanfang Hospital, Southern Medical University, Guangzhou, Guangdong 510515 China

**Keywords:** Serum creatinine, Cystatin C, Glomerular filtration rate, Chronic kidney disease

## Abstract

**Background:**

The aim of this study was to identify the optimal equation that accurately estimates the glomerular filtration rate (GFR) and the chronic kidney disease (CKD) stage in the Chinese population.

**Methods:**

A total of 1296 Chinese patients aged 18–65 years old were enrolled in this study. The estimated GFRs (eGFRs) calculated separately by three Diet in Renal Disease (MDRD) equations and three Chronic Kidney Disease Epidemiology Collaboration (CKD-EPI) equations were compared with the reference GFR (rGFR) measured by the ^99^Tc^m^-DTPA renal dynamic imaging method.

**Results:**

By Bland-Altman analysis, eGFR_cys_ and eGFR_scr_cys_ performed similarly, showing the tightest limits of agreement among the six equations. They also achieved the first and second highest 30% and 50% accuracies. Using a combination of the serum creatinine and cystatin C levels (eGFR_scr_cys_) could improve the bias (−0.3 for eGFR_scr_cys_) of the equation and achieve the highest diagnostic accuracy for renal insufficiency (AUC_60_, 0.953; *P* < 0.05, except for eGFR__MDRD_). All equations predicted stage 3 CKD with moderate accuracy (49.7–51.4%) and stage 5 CKD with good accuracy (90.2–96.4%). For stage 1 CKD, eGFR_cys_ showed a higher percentage of misclassification than the other equations. All equations seemed to perform poorly at predicting stage 2 and 4 CKD, as compared to the other CKD stages. eGFR_scr_cys_ was the best-performing equation in terms of accurate classification of the CKD stage based on the overall performance (kappa value, 0.423).

**Conclusion:**

For a Chinese population, the CKD-EPI_scr_cys_ equation seems more suitable for estimating the GFR than the other equations. Each equation had its own advantages in predicting different CKD stages.

## Background

The prevalence of chronic kidney disease (CKD) is estimated to be 8–16% worldwide, generating a heavy economic impact on society in both developed and undeveloped countries [1]. The glomerular filtration rate (GFR) has generally been considered the vital indicator for predicting overall renal function. Therefore, accurate estimation of the GFR is important for assessing the severity and progression of CKD.

Serum creatinine is an easily measurable and widely available marker of renal function. However, its levels are affected by multiple factors, such as muscle mass, weight, gender, etc. [2]. Cystatin C is a nonglycosylated low-molecular-weight protein that is in the cystatin superfamily of cysteine protease inhibitors. It is produced at a constant rate by all nucleated cells and is freely filtered by the glomerulus. Although the serum cystatin C level serves as a valuable tool for early detection of renal dysfunction, it has been reported to be influenced by age, gender, body mass index, smoking status, the C-reactive protein level, nephritis, and hypertension [3–6].

The estimated GFR (eGFR), calculated by different equations, is commonly used for clinical care and research. The Modification of Diet in Renal Disease (MDRD) equation, initiated in 1999 and based on the serum creatinine level, is still applied clinically after several modifications [7, 8]. Recently, new equations such as the Chronic Kidney Disease Epidemiology Collaboration (CKD-EPI) equations based on cystatin C and/or serum creatinine have been recommended for clinical applications [9, 10]. Some reports showed an improved accuracy of the eGFR using the cystatin C-based eq. [11, 12]. However, it is still controversial whether cystatin C-based GFR-estimating formulae are superior to serum creatinine-based ones [6].

Until now, limited data are available on the comparison of six GFR-estimating equations (CKD-EPI_scr_, CKD-EPI_cys_, CKD-EPI_scr_cys_, abbreviated MDRD, Chinese MDRD, and original MDRD) in Chinese CKD patients. The aim of this study was to identify the equation that is the most accurate and acceptable for predicting the GFR and the CKD stage in a large Chinese population in a single center.

## Methods

### Subjects

A total of 7676 Chinese participants who underwent GFR measurement using ^99^Tc^m^-diathylenetriamine pentaacetic acid (^99^Tc^m^-DTPA) scintigraphy from January 2009 to March 2016 in Nanfang Hospital, China, were observed. The following exclusion criteria were used: 1) younger than 18 years old or older than 65 years old (*n* = 1124); 2) obstructive nephropathy (*n* = 4060); 3) solitary kidney or a single kidney (*n* = 6); 4) urinary inflammation (*n* = 58); 5) acute renal insufficiency or injury (*n* = 10); 6) any history of malignancy or kidney surgery (*n* = 918); 7) hyperthyroidism (*n* = 4); 8) use of antibacterial agents within 2 weeks (*n* = 145); 9) malignant hypertension (*n* = 44). A total of 1307 patients were screened in the preliminary study, and 11 out of 1307 patients failed to meet diagnostic criteria of CKD who were excluded from the study. Finally, a total of 1296 eligible patients were enrolled in this study. The diagnostic criteria of CKD are in accordance with the K/DOQI practice guidelines. Written informed consent was obtained from each subject prior to participation. This study was approved by the Ethics Committee of the Nanfang Hospital of Sothern Medical University.

### Measurement of reference GFR (rGFR) and CKD classification

The rGFR was measured by nuclear medicine techniques. Participants were well hydrated before examination. ^99^Tc^m^-DTPA (radiochemical purity, >95%; percentage of ^99^Tc^m^-DTPA bound to plasma protein, <5%) was provided by Guangzhou Atomic Isotope Hi-Tech Pharmaceutical Co., Ltd., China.

CKD was classified into five stages based on the rGFR values as follows [13]: stage 1, rGFR ≥90 mL/min/1.73 m^2^; stage 2, 60 mL/min/1.73 m^2^ ≤ rGFR <90 mL/min/1.73 m^2^; stage 3, 30 mL/min/1.73 m^2^ ≤ rGFR <60 mL/min/1.73 m^2^; stage 4, 15 mL/min/1.73 m^2^ ≤ rGFR <30 mL/min/1.73 m^2^; stage 5, rGFR <15 mL/min/1.73 m^2^. Renal insufficiency was defined as rGFR <60 mL/min/1.73 m^2^.

### Measurement of serum creatinine and cystatin C levels

Serum creatinine and cystatin C levels were measured in the fasting state by a sarcosine oxidase assay kit (Sichuan Maker Biotechnology Co., Ltd., China) and an immunoturbidimetric assay kit (Beijing Leadman Biotechnology Co., Ltd., China) on an Olympus AU5421® analyzer, respectively.

The eGFR was calculated based on the serum creatinine and/or cystatin C levels using six estimating equations (Table 1).

**Table 1:** Different equations for estimation of GFR

Subjects	Gender	Scr (mg/dL)	Scys (mg/L)	Equation (mL/min/1.73 m^2^)
CKD-EPI_scr_ (eGFR_scr_)	female	≤0.7		141 × (Scr/0.7)^-0.329^ × 0.993^age^ × 1.018
		>0.7		141 × (Scr/0.7)^-1.209^ × 0.993^age^ × 1.018
	male	≤0.9		141 × (Scr/0.9)^-0.411^ × 0.993^age^
		>0.9		141 × (Scr/0.9)^-1.209^ × 0.993^age^
CKD-EPI_cys_ (eGFR_cys_)	female		≤0.8	133 × (Scys/0.8)^-0.499^ × 0.996^age^ × 0.932
			>0.8	133 × (Scys/0.8)^-1.328^ × 0.996^age^ × 0.932
	male		≤0.8	133 × (Scys/0.8)^-0.499^ × 0.996^age^
			>0.8	133 × (Scys/0.8)^-1.328^ × 0.996^age^
CKD-EPI_scr___cys_ (eGFR_scr___cys_)	female	≤0.7	≤0.8	130 × (Scr/0.7)^-0.248^ × (Scys/0.8)^- 0.375^ × 0.995^age^
			>0.8	130 × (Scr/0.7)^-0.248^ × (Scys/0.8)^- 0.711^ × 0.995^age^
		>0.7	≤0.8	130 × (Scr/0.7)^-0.601^ × (Scys/0.8)^- 0.375^ × 0.995^age^
			>0.8	130 × (Scr/0.7)^-0.601^ × (Scys/0.8)^- 0.711^ × 0.995^age^
	male	≤0.9	≤0.8	135 × (Scr/0.9)^-0.207^ × (Scys/0.8)^- 0.375^ × 0.995^age^
			>0.8	135 × (Scr/0.9)^-0.207^ × (Scys/0.8)^- 0.711^ × 0.995^age^
		>0.9	≤0.8	135 × (Scr/0.9)^-0.601^ × (Scys/0.8)^- 0.375^ × 0.995^age^
			>0.8	135 × (Scr/0.9)^-0.601^ × (Scys/0.8)^- 0.711^ × 0.995^age^
abbreviated _MDRD (eGFR_a___MDRD_)				186 × (Scr)^-1.154^ × (age)^-0.203^ × (×0.742 if female)
Chinese _MDRD (eGFR_c___MDRD_)				175 × (Scr)^-1.234^ × (age)^-0.179^ × (×0.79 if female)
original MDRD (eGFR__MDRD_)				186 × (Scr)^-1.154^ × (age)^-0.203^ × (×0.742 if female) × (×1.233 if Chinese)

*Scr* serum creatinine, *Scys* serum cystatin C

### Statistical analysis

Statistical analysis was performed using SPSS20.0 (SPSS Inc., Somers, NY, USA) and MedCalc13.0 (MedCalc, Mariakerke, Belgium). Quantitative data were tested for homogeneity of variance by the Kolmogorov-Smirnov one-sample test. Bland-Altman analysis was used to determine the agreement between the rGFR and eGFR values, which were calculated by different equations. The receiver operating characteristic (ROC) curve was used to determine the diagnostic power at predicting the renal insufficiency (ROC_60_) by the six different equations, with the results reported as the areas under the ROC curve (AUC_60_), sensitivity, and specificity. Kappa statistics were used to evaluate the agreement between stage classification from the rGFR values and from the eGFR values calculated by different equations, with the following interpretations: poor agreement (0–0.20), slight agreement (0.21–0.40), moderate agreement (0.41–0.60), good agreement (0.61–0.80), and excellent agreement (0.81–1.0). The paired sample t test was carried out to evaluate inter-group differences. Differences with *P* < 0.05 were considered statistically significant.

## Results

### Participant characteristics

A total of 1296 participants aged 45.0 (35.0, 55.0) years old were enrolled, including 814 males and 482 females. The mean rGFR was 46.8 (29.8, 68.3) mL/min/1.73 m^2^, whereas the mean eGFR varied based on the different calculation formulae and ranged from 40.1 (19.2, 69.3) mL/min/1.73 m^2^ to 52.0 (21.6, 88.3) mL/min/1.73 m^2^. The basic characteristics of the participants are shown in Table 2.

**Table 2:** Baseline characteristics of the participants

Variable	Value
Age, years	45.0 (35.0, 55.0)
Gender, male, n (%)	814 (62.8%)
Weight, kg	62.0 (54.0, 70.0)
Height, cm	165.0 (158.0,170.0)
Body surface area, m^2^	1.7 ± 0.2
Body mass index, Kg/m^2^	22.9 (20.8, 25.4)
Serum creatinine, mg/dL	1.5 (1.0, 3.2)
Serum cystatin C, mg/L	1.7 (1.1, 2.9)
rGFR, mL/min/1.73 m^2^	46.8 (29.8, 68.3)
eGFR, mL/min/1.73 m^2^	
eGFR_scr_	49.7 (19.6, 85.8)
eGFR_cys_	40.1 (19.2, 69.3)
eGFR_scr___cys_	44.7 (18.6, 76.5)
eGFR_a___MDRD_	48.9 (20.1, 81.0)
eGFR_c___MDRD_	49.4 (19.1, 85.2)
eGFR__MDRD_	52.0 (21.6, 88.3)
rGFR based on CKD stage, mL/min/1.73 m^2^	
Stage 1 (*n* = 104)	101.4 (96.2, 111.2)
Stage 2 (*n* = 331)	73.2 ± 8.6
Stage 3 (*n* = 529)	43.5 (37.0, 51.5)
Stage 4 (*n* = 220)	23.6 (19.5, 27.0)
Stage 5 (*n* = 112)	10.8 (8.5, 13.2)

*eGFR* estimated glomerular filtration rate, *rGFR* reference glomerular filtration rate, *CKD* chronic kidney disease, *MDRD* modification of diet in renal disease, *CKD-EPI* chronic kidney disease epidemiology collaboration

### Performance of the six equations compared with the rGFR

**Fig. 1:**
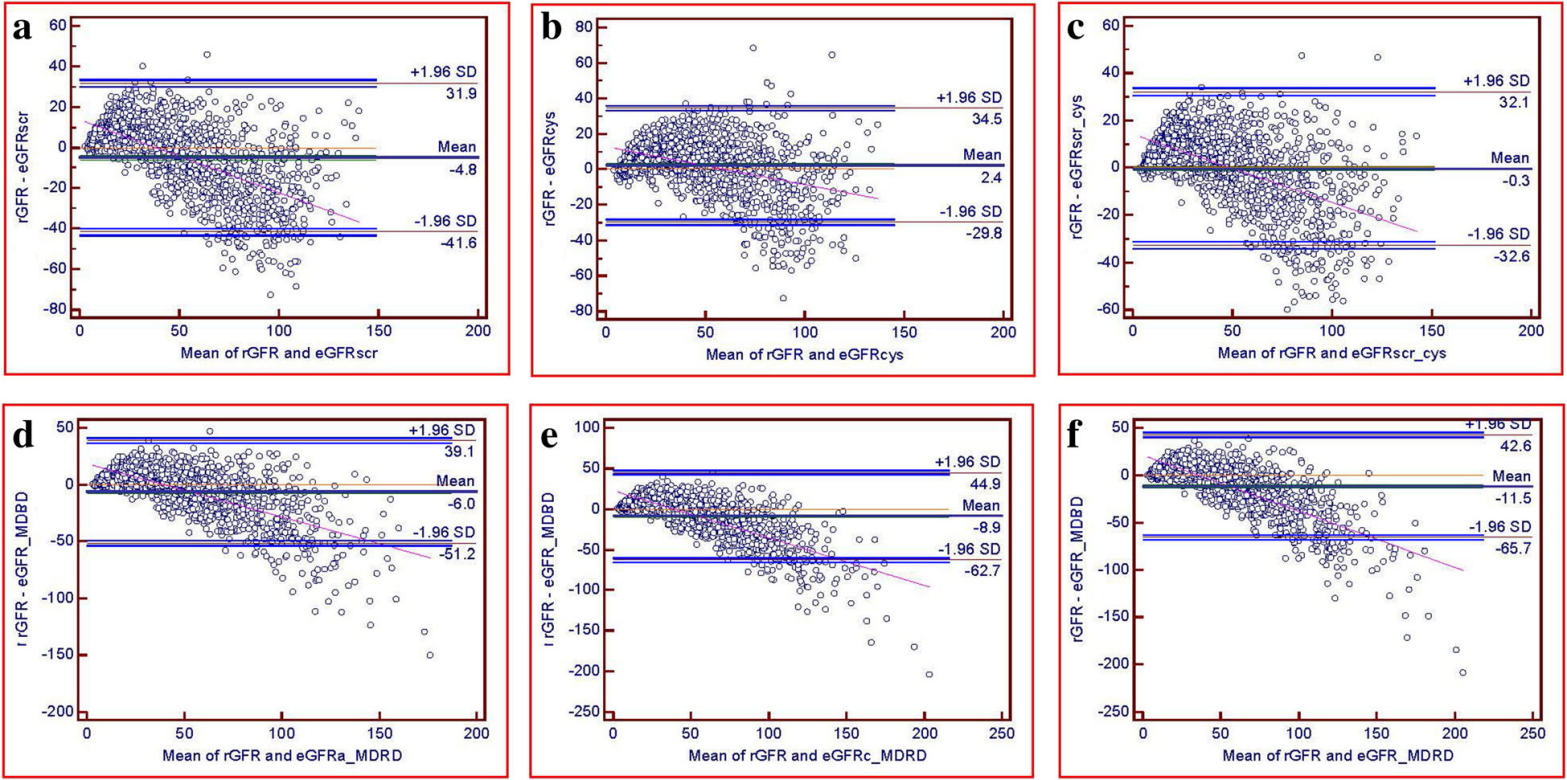
Bland-Altman plots of the rGFR and eGFR (mL/min/1.73 m^2^). eGFR_cys_ and eGFR_scr_cys_ performed similarly, showing the tightest limits of agreement among the six equations. eGFR_scr_cys_ showed the least bias among the six equations. eGFR_scr_ (**a**), eGFR_cys_ (**b**), eGFR_scr_cys_ (**c**), eGFR_a_MDRD_ (**d**), eGFR_c_MDRD_ (**e**), and eGFR__MDRD_ (**f**)

The agreement or disagreement between the eGFR values and the rGFR values was analyzed by Bland-Altman plots (Fig. 1). According to these plots, the limits of the regression lines varied by each equation and were 73.5 for eGFR_scr_, 64.3 for eGFR_cys_, 64.7 for eGFR_scr_cys_, 90.3 for eGFR_a_MDRD_, 107.6 for eGFR_c_MDRD_, and 108.3 for eGFR__MDRD_. The eGFR_cys_ and eGFR_scr_cys_ equations performed similarly, showing the tightest limits of agreement among the six equations. The biases of eGFR_cys_ and eGFR_scr_cys_ (2.4 and −0.3, respectively) were much less than those of eGFR_scr_, eGFR_a_MDRD_, eGFR_c_MDRD_, and eGFR__MDRD_ (−4.8, −6.0, −8.9, and −11.5, respectively). Thus, the equations based on serum creatinine, eGFR_scr_, eGFR_a_MDRD_, eGFR_c_MDRD_, and eGFR__MDRD_, had poor agreement with the rGFR (eGFR_c_MDRD_ and eGFR__MDRD_ in particular). Using a combination of serum creatinine and cystatin C levels could improve the bias (−0.3 for eGFR_scr_cys_) of the equation.

### Diagnostic performance of the six equations for predicting renal insufficiency

The diagnostic performance for predicting renal insufficiency based on the six equations is summarized in Table 3. The AUC_60_ at a cutoff point of 59.6 mL/min/1.73 m^2^ in eGFR_scr_cys_ achieved the highest value (0.953), with a sensitivity of 87.6% and a specificity of 89.1%, suggesting the highest diagnostic accuracy for predicting renal insufficiency (*P* < 0.05 vs. the others, except for eGFR__MDRD_). The optimal cutoff point of eGFR_cys_ for predicting renal insufficiency was 46.8 mL/min/1.73 m^2^, with a sensitivity of 93.6%, a specificity of 81.0%, and an AUC_60_ of 0.945. A revised cutoff value of eGFR_cys_ to 60.6 mL/min/1.73 m^2^ led to an improved specificity of 91.5% and a decreased sensitivity of 77.1%.

**Table 3:** Diagnostic performance of the six equations for predicting renal insufficiency (mL/min/1.73 m^2^)

Equation	eGFR_scr_	eGFR_cys_	eGFR_scr___cys_	eGFR_a___MDRD_	eGFR_c___MDRD_	eGFR__MDRD_
Cutoff Value	62.8	46.8	59.6	59.3	63.2	69.0
AUC_60_	0.948*	0.945*	0.953	0.945*	0.948*	0.951^●^
95% CI	0.934–0.959	0.931–0.957	0.940–0.964	0.933–0.958	0.934–0.959	0.938–0.962
Youden index J	0.745	0.745	0.767	0.743	0.748	0.760
Sensitivity	89.0	93.6	87.6	89.9	88.7	86.9
Specificity	85.5	81.0	89.1	84.5	86.1	89.1
Adjustment Cutoff Value	60.4	60.6	60.2	60.0	60.3	59.8
Sensitivity	90.3	77.1	86.7	88.1	89.9	92.2
Specificity	84.1	91.5	89.1	84.7	83.9	81.7

^●^ *P =* 0.4206 compared with eGFR_scr_cys_

*P < 0.05 compared with eGFR_scr_cys_

### Misclassification of CKD stages by the six equations

All equations had a high accuracy (range, 90.2–96.4%) for the diagnosis of stage 5 CKD; whereas all of them exhibited a moderate accuracy for the diagnosis of stage 3 CKD (Table 4). In stage 1 CKD, eGFR_cys_ showed a higher percentage of misclassification than the other equations. Although eGFR_scr_cys_ exhibited the highest accuracy for estimating stage 2 CKD (49.8%), all equations seemed to perform poorly at predicting stage 2 and 4 CKD, as compared to the other CKD stages. In addition, based on the overall performance, eGFR_scr_cys_ had the highest kappa value (0.423), compared to the other five equations, suggesting that eGFR_scr_cys_ might be the best-performing equation in terms of accurate classification of the CKD stage.

**Table 4:** CKD stage classification based on eGFR estimation by the six equations (*n* = 1296)

Equation	CKD stage based on rGFR					Kappa
	Stage 1	Stage 2	Stage 3	Stage 4	Stage 5	
eGFR_scr_	98 (94.2%)	121 (36.6%)	263 (49.7%)	83 (37.7%)	107 (95.5%)	0.392
eGFR_cys_	78 (75.0%)	152 (45.9%)	266 (50.3%)	101 (45.9%)	103 (92.0%)	0.406
eGFR_scr_cys_	88 (84.6%)	165 (49.8%)	272 (51.4%)	79 (35.9%)	108 (96.4%)	0.423
eGFR_a_MDRD_	93 (89.4%)	137 (41.4%)	269 (50.9%)	87 (39.5%)	107 (95.5%)	0.407
eGFR_c_MDRD_	98 (94.2%)	116 (35.0%)	264 (49.9%)	81 (36.8%)	108 (96.4%)	0.387
eGFR__MDRD_	99 (95.2%)	109 (32.9%)	264 (49.9%)	95 (43.2%)	101 (90.2%)	0.388

### Accuracy of the six eGFRs and the rGFR

Among the six equations, eGFR_scr_cys_ had the smallest bias, whereas eGFR_cys_ exhibited the highest 30% accuracy and 50% accuracy (Table 5).

**Table 5:** Comparison of bias and accuracy between the eGFR and rGFR

Equation	Bias (mL/min/1.73 m^2^)	Bias of 95% CI	30% accuracy	50% accuracy
eGFR_scr_*	−4.8	−5.8, −3.8	51.2%	77.6%
eGFR_cys_*	2.4	1.5, 3.3	60.3%	86.4%
eGFR_scr___cys_^▲^	−0.3	−1.2, 0.6	57.4%	83.7%
eGFR_a___MDRD_*	−6.0	−7.3, −4.8	52.1%	76.4%
eGFR_c___MDRD_*	−8.9	−10.4, −7.4	47.1%	71.3%
eGFR__MDRD_*	−11.5	−13.0, −10.0	49.0%	73.5%

*Compared with rGFR, *P* = 0.000

▲Compared with rGFR, *P* = 0.550

## Discussion

Each GFR-estimating equation has its own advantages for different stages of impaired renal function. In addition, their performances are affected by various factors. First, the serum creatinine level is determined by different methods. Compared with the Jaffe method, the enzymatic method is less affected by external factors [14–16]. A previous study has shown a significantly higher accuracy for the GFR-estimating equation using the enzymatic method to measure creatinine than that measured by the picric acid method when the rGFR is ≥60 mL/min/1.73 m^2^ [17]. Second, the patients had different ages. The research performed by Roberts et al. showed that the MDRD equation overestimates the renal function in different age groups, which does not become apparent until after 65 years of age [18]. Thus, the role of age in GFR estimation should be taken into consideration, and the elderly participants (over 65 years old) need to be observed separately. Therefore, this study only included patients aged 18–65 years old in order to minimize the possible bias of the study. Third, racial factors can affect the results. A meta-analysis has revealed that cystatin C has a better diagnostic value for CKD in the West than in Asia [19], suggesting the performance of the equation differs in different racial and ethnic populations.

The inulin clearance rate has been considered as the gold standard, but it is an impractical method for estimating renal function, probably due to its costly, cumbersome features. Thus, radioisotopic methods, such as ^99^Tc^m^-DTPA, are commonly used in clinical applications [20, 21]. Serum creatinine and cystatin C represent two other indicators for predicting renal function. Cystatin C seems to perform better at predicting an early decrease in renal function [22–25], particularly in the elderly [6], whereas serum creatinine is insensitive until the impairment is 50% or more [26]. Our previous study revealed that the serum cystatin C measurement is more sensitive than that of serum creatinine for detecting an early decline in the rGFR [22]. In the present study, the Bland-Altman plots showed that eGFR_cys_ and eGFR_scr_cys_ had similar low limits of agreement among the six equations, revealing higher agreement of cystatin C-based equations with the rGFR than the other four serum creatinine-based equations.

Accurate CKD stage classification facilitates the successful management of such patients. The performances of the GFR-estimating equations based on either serum creatinine or serum cystatin C vary with race/ethnicity [7, 19]. Thus, it is extremely important to identify the best-performing equation for CKD stage classification in specific populations. Previously, we found that the CKD-EPI equation based on the serum creatinine level exhibited a better performance than the MDRD equation in estimating the GFR in Chinese diabetics [27]. In the present study, all six equations achieved high classification accuracy for stage 5 CKD (≥90.2%), no matter which serum creatinine or cystain C-based equation was used. However, their diagnostic efficiency differed greatly in CKD at stages 1–4. eGFR_cys_ had a low diagnostic accuracy in stage 1 CKD at a cutoff value of 46.8 mL/min/1.73 m^2^, which is lower than that used clinically, suggesting that cystatin C might underestimate mild renal dysfunction. Since serum creatinin- and cystatin C-based equations are most applicable in different CKD stages [6], it has been reported that equations based on a combination of creatinine and cystatin C perform better than those equations based on creatinine or cystatin C alone [9, 28]. Among these equations, eGFR_scr_cys_ had the highest AUC_60_ among the six equations in the ROC_60_ analysis (*P* < 0.05 vs. the others, except for eGFR__MDRD_), and it also achieved the top accuracy for overall CKD classification (kappa value, 0.423). These findings are consistent with those from Ying Zhu et al. [29] and reveal that GFR-estimating equations based on the combination of serum creatinine and cystatin C levels may improve diagnostic efficiency for renal function.

## Conclusions

This study has one particular strength. Considering that racial factors can affect the results, this study focused on data from a Chinese population for the purpose of identifying an appropriate GFR equation for the Chinese population. Also, this study only included Chinese patients aged 18–65 years old to minimize age-related bias of the study effectively.

This study also has some limitations. First, this was a retrospective, single-center study in China. Thus, caution must be used when generalizing the results of this study in a different population. Second, this study included adult patients aged from 18 to 65 years old. Considering the age-related decline in the GFR, we cannot be sure of the relevance of the results among children or elderly patients. Third, the role of some unmeasured factors (diet, muscle mass, etc.) that could have possibly influenced the observed association cannot be entirely ruled out. Forth, although ^99^Tc^m^-DTPA renal dynamic imaging has been widely as reference standard for clinical evaluation of renal function, it still has its disadvantages. Some researchers believe that ^99^Tc^m^-DTPA renal dynamic imaging may underestimate the true GFR [30] because a very small part of ^99^Tc^m^-DTPA bounds to plasma proteins, although this is only speculated theoretically, not on the basis of pathological biopsy, and is usually neglected.

In conclusion, the CKD-EPI equations had higher agreement with the rGFR than the MDRD equations. Our study also found that the CKD-EPI_scr_cys_ equation achieved the top accuracy for overall CKD classification in the Chinese population. Compared with CKD-EPI_scr_ and CKD-EPI_cys_, the use of the combination of serum creatinine and cystatin C (CKD-EPI _scr_cys_) levels could improve the bias of the equation and achieve a higher diagnostic accuracy for renal insufficiency. Each equation had its own advantages in predicting different CKD stages and needs further research.

## Abbreviations

^99^Tc^m^-DTPA: ^99^Tc^m^-diathylenetriamine pentaacetic acid
AUC: The areas under the ROC curve
CKD: Chronic kidney disease
CKD-EPI: Chronic Kidney Disease Epidemiology Collaboration
eGFR: Estimated GFR
GFR: Glomerular filtration rate
MDRD: Modification of Diet in Renal Disease
rGFR: Reference GFR
ROC: Receiver operating characteristic
